# Effect of Parameters of Pool Geometry on Flow Structure in Nature-like Fishway

**DOI:** 10.3390/ijerph19159389

**Published:** 2022-07-31

**Authors:** Qiaoyi Hu, Xiaogang Wang, Long Zhu, Shuai Du, Feifei He

**Affiliations:** 1 Nanjing Hydraulic Research Institute, Nanjing 210029, China; huqiaoyi1995@163.com (Q.H.); ffhe@foxmail.com (F.H.); 2 Anhui Provincial Group Limited for Yangtze-to-Huaihe Water Diversion, Hefei 230000, China; dushuai@ahyjjh.com.cn

**Keywords:** nature-like fishway, permeability ratio, offset ratio, bottom slope, pool length

## Abstract

Many uncertainties such as variable irregular structure and complex flow conditions bring difficulties to the design of a nature-like fishway. This study defines the main factors and parameters affecting flow conditions such as permeability ratio, offset ratio, bottom slope and pool length to simplify and generalize the irregular geometry of the nature-like fishway. According to the engineering requirements of the Mopiling nature-like fishway, the effect of the above parameters of pool geometry on the flow structure is investigated through a 3D turbulent numerical simulation, and the parameter thresholds are summarized according to the optimization of the flow conditions. The results show that under the same conditions, the maximum velocity of the control section increases with the increase in permeability ratio, bottom slope and pool length, and the offset ratio has limited effect on the maximum velocity of the control section. It is recommended that when the bottom slope is 1/250 and the pool length is 10 m, the permeability ratio should not be greater than 0.30 and the offset ratio should be located between 0.15 and 0.60. When the bottom slope is adjusted to 1/200, it is recommended to control the permeability ratio below 0.20, the offset ratio between 0.30 and 0.60, and the pool length can be adjusted to 8 m. Within the above threshold range of the design parameters, the maximum velocity in the fishway can be basically controlled at about 1.0 m/s. The mainstream in the pool is clear and the flow pattern is good, which can basically satisfy the requirements of fish passing. The relevant design parameters and optimization strategies can provide reference for similar projects.

## 1. Introduction

The construction of water conservancy projects such as locks and dams can meet the requirements of flood control, irrigation, power generation, etc. While generating huge social and economic benefits, they also form a barrier to fish in rivers, hindering the genetic exchange of fish upstream and downstream of the dam site and destroying the ecological environment on which fish and other aquatic organisms rely for survival. The fishway is responsible for opening up the fish migration channel and safeguarding the aquatic ecological environment of the watershed. The fish are sensitive to the water flow conditions in the fishway, and the hydraulic characteristics of the fishway are also complicated, so it is necessary to conduct hydraulics research on the fishway to ensure the effect of fish passing [1,2,3,4,5,6].

There are two types of fishways: technical fishways and nature-like fishways. At present, many scholars have studied the technical fishway, especially for the vertical-slot fishway; they have mainly studied the flow characteristics of the common pool or the turning pool [7,8,9], and analyzed the effects of pool geometry changes such as the length and width of the pool, the layout of the vertical slots, the bottom slope and the macro-roughness on the flow conditions [10,11,12,13,14]. For the complex turbulent flow in the vertical-slot fishway, researchers also used more sophisticated turbulent models such as the detached eddy simulation and the large eddy simulation to study the velocity and eddy [15,16,17,18]. A good vertical-slot fishway design should ensure that the hydraulic characteristics of the pool are compatible with the swimming ability of the fish. Researchers observed the fish upstreaming process in the vertical-slot fishway, analyzed the motion characteristics, revealed that the fish movement was related to the preference range of hydraulic characteristics such as velocity and turbulent kinetic energy [19,20,21,22,23], and simulated the fish trajectory using a numerical model [24,25]. Generally, the structure of a technical fishway is relatively regular, which is convenient for design. However, the internal flow structure and flow pattern of the pool are slightly single, which is only suitable for some special fishes. However, in practical engineering, there are various passing objects that need to be protected in rivers. Among them, the body shape is quite different, and the swimming ability is also different. Especially, the swimming behavior and energy cost-recovery of fish passing through velocity barriers are very complex [26,27,28]. The construction of fishway needs to use an interdisciplinary approach, considering aquatic biology and hydraulics. How to design a fishway that can consider a variety of passing objects and improve the efficiency of fish passing is the key and difficult point of the research.

A nature-like fishway is a broad term for several styles of structures such as bottom ramps and slopes, bypass channels and fish ramps, with the chosen construction material corresponding to what is usually present in rivers under natural conditions. Compared with traditional technical fishways, the flow conditions in nature-like fishways tend to be more diversified, providing both fish passage and habitat for a variety of aquatic organisms [29,30,31,32]. The nature-like fishway optimizes the construction material and structure form ecologically, pays more attention to the overall coordination with the surrounding environment, and can form an ecological-landscape corridor [33]. For hydraulic-design aspects of nature-like fishways, Acharya et al. [34] studied the flow field around the single geometric flow obstruction of different shapes and gave recommendations for the spacing of isolated roughness elements in the fishway. He et al. [35] compared the hydraulic characteristics in the fishway of the rocky ramp type under aligned slot arrangement and staggered slot arrangement. Baki et al. [36] investigated the hydraulic characteristics of a rock-weir fishway with and without passage notches. Zhang et al. [37] studied the cross-section form with multi-stage velocity in the fishway by adjusting the arrangement of multiple groups of wild stones. Hu et al. [38] and Ma et al. [39] studied the flow conditions under the structure combination of a bottom outlet or surface outlet and a vertical slot. Li et al. [40] compared the hydraulic characteristics of fishways with cobblestone weirs and fishways with impermeable weirs, and analyzed the effect of cobblestone weirs’ porosity on flow conditions. The nature-like fishway often has a slow bottom slope, a long route and many changes; it is easy to accumulate energy at all levels of the pools. Xu et al. [41] established the overall physical model of the nature-like fishway, and demonstrated the overall and local flow conditions and the local water supplement measures. The nature-like fishway is designed according to the site topography and natural materials to simulate the natural river as much as possible. Many uncertainties such as the variable irregular structure and the complex flow conditions bring difficulties to the design of the nature-like fishway. Fewer nature-like fishways have been built and are under construction in China, and there is a lack of sufficient construction experience and post-evaluation of fish-passing effects. Due to the complexity of the subject, there is a lack of simple, effective and well-developed research methods or design guidelines for the nature-like fishway [41].

This study defines the main factors and parameters such as the permeability ratio, offset ratio, bottom slope and pool length to simplify and generalize the irregular geometry of the nature-like fishway. According to the engineering requirements of the Mopiling nature-like fishway, in the study, we carried out a three-dimensional turbulent numerical simulation. The effect of parameters of pool geometry on the flow structure was investigated, and the design parameter thresholds meeting the engineering requirements were obtained according to the optimization of the flow conditions, which can provide a theoretical reference for the construction of the nature-like fishway.

## 2. Materials and Methods

### 2.1. Study Sites

The Xinjiang River is located in the northeastern part of Jiangxi Province, China, and it is rich in fish resources and has many economic fish species. Mopiling hydrojunction is part of the Bazizui hydrojunction on the Xinjiang River; the construction will form a barrier to fish in the hydrojunction area and even the entire mainstream of the Xinjiang River, causing damage to the ecological environment. In order to open up the fish migration channel between the upstream and downstream of the Mopiling hydrojunction, it is proposed to build a nature-like fishway (the bypass channel) with abundant construction space and good layout conditions.

The main protecting targets of the fishway are economic fish such as the “four great fish” and the original rare fish such as shad in the Xinjiang River. The design velocity of the fishway refers to the maximum velocity of the control section of the fishway under the design water level difference. It is generally believed that the ability of fishes to overcome the velocity increases with their body length, so in the preliminary design, the design velocity of the fishway can be selected according to the body length of the main fish-passing objects. According to the data, for Cyprinidae fish with a body length greater than 30 cm, the design velocity of the fishway is usually 1.0 m/s~1.2 m/s, and the favorite velocity of the shad is 0.7 m/s~1.0 m/s. So, the design velocity of this fishway is determined to be 0.7 m/s~1.2 m/s; the study controlled the maximum velocity of the fishway at about 1.0 m/s.

The design strategy of the Mopiling nature-like fishway is that the total length and slope of the fishway are determined by considering the topography. The interior of the fishway basically adopts a trapezoidal section with the partition walls set at every distance along the fishway, constructed with natural materials such as rock to form the structure of the vertical slots. The main season of the fish migration is from April to July every year; during the season, the design’s water level at the fishway outlet is 18.0 m, and the design’s low water level at the inlet is 12.95 m. The total length of the effective climbing section of the fishway is determined to be about 1265 m, with an average slope of 1/250.

### 2.2. Generalization Strategy and Numerical Model of Nature-Like Fishways

There are variable cross-sectional patterns and many influencing factors for nature-like fishways. The study could not be carried out effectively if all the complex factors were taken into account. In order to understand its basic hydrodynamic characteristics, this study simplified and generalized the nature-like fishway by not considering other factors such as possible bends, resting pools, shoals and deep pools in the bypass channel, but only considering the linear section and the basic unit of the fishway pool.

**Figure 1:**
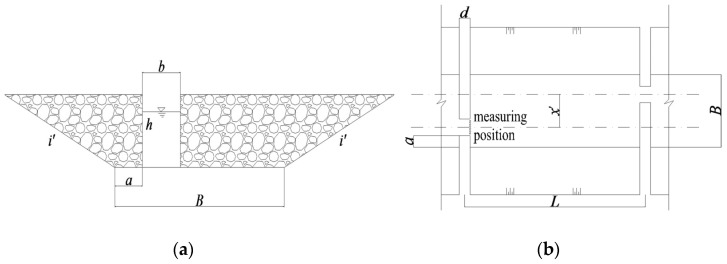
Schematic of fishway model. (**a**) The cross-section of vertical slot (**b**) The structural arrangement of pool.

The nature-like fishway adopts a trapezoidal arrangement. The partition wall is set at every interval in the fishway to form water blocking and energy dissipation, and the pool is formed between adjacent partition walls. There are several water-permeable areas in the partition wall, and the water-permeable area of adjacent partition walls is arranged on different sides to form the mainstream. In this paper, the irregular permeable area in partition walls is simplified as a rectangle. According to the concept of the narrow permeable zone of the control section in the vertical-slot fishway, the rectangular permeable area in the nature-like fishway is also called “vertical slot”. The general layout of the partition wall and the pool is shown in Figure 1. In the figure, B represents the bottom width of the pool, b represents the width of the vertical slot, $i^{\prime }$ represents the side slope of the fishway, h represents the water depth, a represents the offset distance at the bottom of the short partition wall, d represents the thickness of the partition wall, $x^{\prime }$ represents the distance between the centers of adjacent vertical slots, and *L* represents the length of the pool.

There are two important causes of water flow energy consumption in the fishway: water blockage by the partition wall and mainstream bypass deflection of the pool. Based on these two reasons, the irregular cross-section and the pool geometry is simplified and generalized. The water-permeable area of the partition wall is expressed by the permeability ratio of the control section, referred to as the permeability ratio. Additionally, the mainstream deflection length of the pool is expressed by the offset ratio of the control section of the adjacent pool, referred to as the offset ratio.

There are many factors that affect the flow conditions, based on the generalization above; the main ones are permeability ratio, offset ratio, bottom slope, side slope, bottom width and pool length. Within a certain range, changes in the side slope and bottom width have limited effect on the flow conditions [38,41]. So, the main influencing factors discussed in the study are permeability ratio W, offset ratio *R*, bottom slope S and pool length *L*. The permeability ratio of the control section can be calculated according to Formula (1):

(1) $$W=A^{\prime }/A$$

$A^{\prime }$ is the water flow area of the control section (i.e., the water flow area of the vertical slots in the generalization, $A^{\prime }=h\cdot b$). A is the trapezoidal water flow area of the pool, $A=(B+h/i^{\prime })h$. The offset ratio of the control section of the adjacent pool can be calculated according to Formula (2):

(2) $$R=x^{\prime }/B$$

In the study, the effects of changes in permeability ratio and offset ratio on the velocity of the pool are calculated and analyzed one by one to obtain reasonable thresholds that meet the engineering requirements. On this basis, the effects of changes in bottom slope and pool length on the velocity of the pool are analyzed to further demonstrate the reasonableness of the thresholds of W and R.

In the preliminary design of the Mopiling nature-like fishway, the pool length is 10.0 m, the bottom width is 4.0 m, the bottom slope is 1/250, and the side slope is 1/2. The partition wall is built with artificial gabions or pebbles, 0.6 m-thick, which are porous and rough. In this study, the nature-like fishway is simplified and generalized according to design parameters such as permeability ratio and offset ratio, focusing on the effect of structural changes on the flow conditions, so the boundary conditions of the geometry are modeled as impermeable and treated as wall boundaries without considering the porosity. For the surface of the geometry, the surface roughness is set to 0.01 m in the simulation. The simulation experience shows that the surface roughness has limited influence on the overall flow condition within a certain range, so it is reasonable to set this value. The numerical model of the fishway was constructed including 9 common pools and 15 m flat sections in both the upstream and downstream, with the total length of 120 m.

To date, Reynolds-averaged Navier–Stokes (RANS) turbulence simulation techniques are the most popular alternative for fishway modelling. Compared with other methods, RANS methods have demonstrated a good accuracy and computational cost [9,14,40]. In the study, the RNG *k*-*ε* model and the method of VOF were used. Additionally, the control equations are expressed in the rectangular coordinate system as follows [35,38,39,42]:

(3) $$\frac{\partial \rho }{\partial t}+\frac{\partial \rho u_{i}}{\partial x_{i}}=0$$

(4) $$\frac{\partial \left(\rho u_{i}\right)}{\partial t}+\frac{\partial \left(\rho u_{i}u_{j}\right)}{\partial x_{j}}=-\frac{\partial p}{\partial x_{i}}+\frac{\partial }{\partial x_{j}}\left[\left(\mu +\mu_{t}\right)\left(\frac{\partial u_{i}}{\partial x_{j}}+\frac{\partial u_{j}}{\partial x_{i}}\right)\right]+\rho g_{i}$$

(5) $$\frac{\partial \left(\rho k\right)}{\partial t}+\frac{\partial \left(\rho ku_{i}\right)}{\partial x_{i}}=\frac{\partial }{\partial x_{j}}\left[\alpha_{k}\left(\mu +\mu_{t}\right)\frac{\partial k}{\partial x_{j}}\right]+G_{k}-\rho \varepsilon$$

(6) $$\frac{\partial \left(\rho \varepsilon \right)}{\partial t}+\frac{\partial \left(\rho \varepsilon u_{i}\right)}{\partial x_{i}}=\frac{\partial }{\partial x_{j}}\left[\alpha_{\varepsilon }\left(\mu +\mu_{t}\right)\frac{\partial \varepsilon }{\partial x_{j}}\right]+C_{1\varepsilon }^{*}\frac{\varepsilon }{k}G_{k}-C_{2\varepsilon }\rho \frac{\varepsilon^{2}}{k}$$

(7) $$\frac{\partial F}{\partial t}+u_{i}\frac{\partial F}{\partial x_{i}}=0$$

where ρ is the fluid density, t is the time, $u_{i}$ and $u_{j}$ are the velocity components, $x_{i}$ and $x_{j}$ are the coordinate components, p is the pressure, μ is the dynamic viscosity, $\mu_{t}$ is the eddy viscosity, the equation is $\mu_{t}=\rho C_{\mu }k^{2}/\varepsilon$, $\rho g_{i}$ is the body acceleration, k is the turbulent kinetic energy, ε is the turbulent dissipation, and *F* is the volume fraction of fluid; the other parameters in the equations are:

(8) $$G_{k}=\mu_{t}\left[\frac{\partial u_{i}}{\partial x_{j}}\left(\frac{\partial u_{i}}{\partial x_{j}}+\frac{\partial u_{j}}{\partial x_{i}}\right)\right]$$

(9) $$C_{1\varepsilon }^{*}=C_{1\varepsilon }-\frac{\eta (1-\eta /\eta_{0})}{1+\beta \eta^{3}}$$

(10) $$\eta =\frac{k}{\varepsilon }\sqrt{\left(2E_{ij}\cdot E_{ij}\right)}$$

(11) $$E_{ij}=\frac{1}{2}\left(\frac{\partial u_{i}}{\partial x_{j}}+\frac{\partial u_{j}}{\partial x_{i}}\right)$$

Empirical constants in the equation: $C_{\mu }=0.0845$, $\alpha_{k}=\alpha_{\varepsilon }=1.39$, $C_{1\varepsilon }=1.42$, $C_{2\varepsilon }=1.68$, $\eta_{0}=4.38$, and β=0.012.

**Figure 2:**
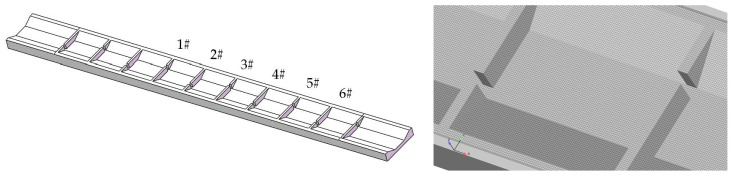
The geometry and mesh.

The calculation area is meshed with 1.73 million hexahedral cells for reconstructing the geometry. The geometry and mesh are shown in Figure 2. Pressure boundary conditions are used for the inlet, outlet and top surface of the numerical model. The water depths of the inlet and outlet are both controlled as 1.0 m. The relative pressure and the volume fraction of fluid at the top boundary of the fishway are both set to 0, indicating that there is only atmosphere. The model set the initial water level and adopted the adaptive time step for calculation, with the initial time step set to 0.01 s, solved by transients until the flow state reaches steadiness and convergence.

### 2.3. Model Validation

**Figure 3:**
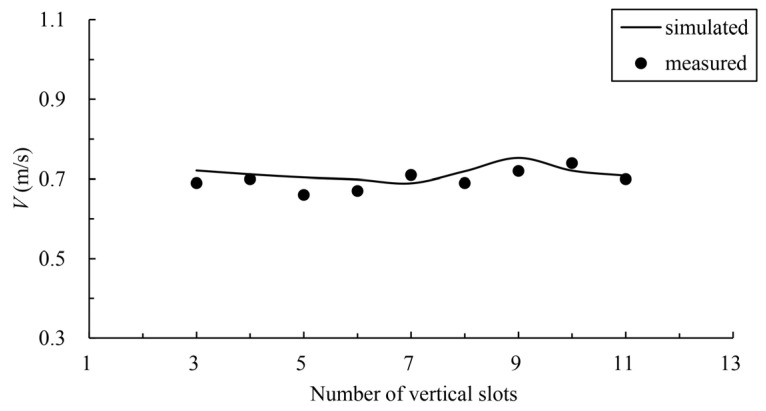
Comparison of simulated and measured maximum velocities among vertical slots.

**Figure 4:**
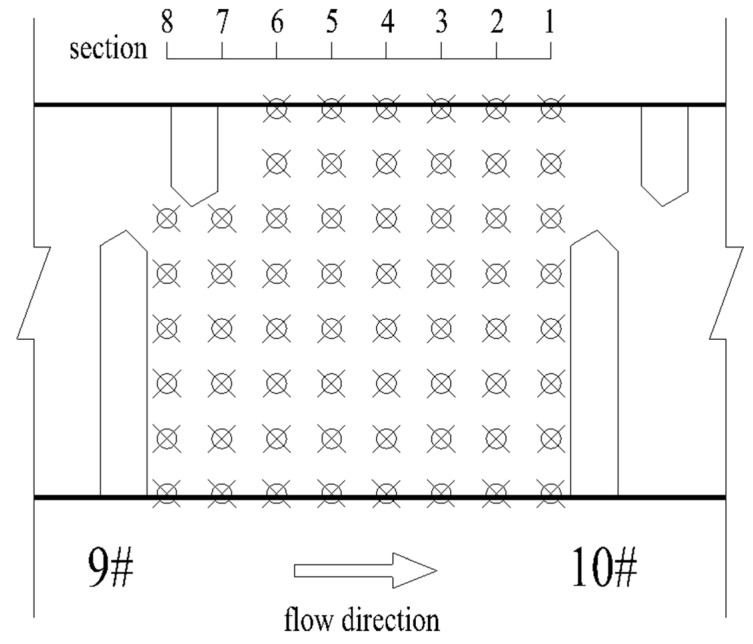
Distribution of measuring points in a typical pool.

**Figure 5:**
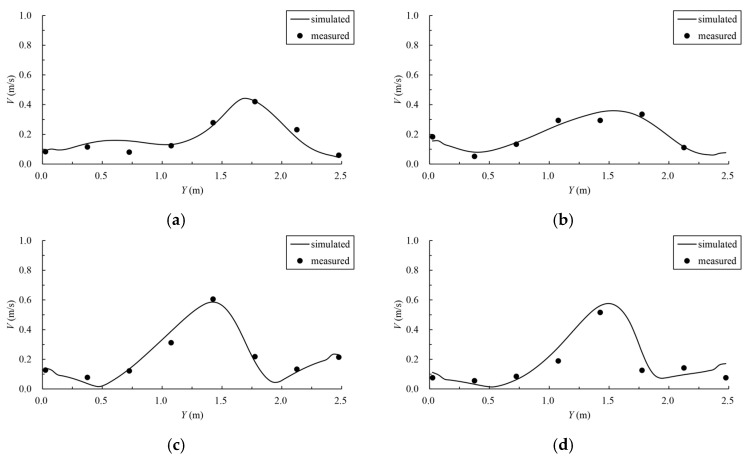
Comparison of simulated and measured velocities in the pool. (**a**) Section 1; (**b**) Section 2; (**c**) Section 5; (**d**) Section 6.

In order to verify the velocity distribution in the fishway pool, the numerical model is verified by using the data of a physical model test of another fishway. The common pool length is 3.0 m, the pool width is 2.5 m, the vertical-slot width is 0.4 m, the water depth is 1.5 m, and the bottom slope is 1/100. The geometric scale of the physical model is 5. The simulation area includes 11 common pools and one resting pool, including 6 pools upstream of the resting pool and 5 pools downstream, with a total of 13 vertical slots (numbered from upstream to downstream 1–13). The study verified the maximum flow velocity of the vertical slot along the fishway from 3 to 11, and the verification results are shown in Figure 3. The velocity measurement data of a typical pool were selected for further verification, the distribution of measuring points is shown in Figure 4, and the comparison of the simulated and measured values is shown in Figure 5, where Y indicates the width direction of the pool. The validation results show that the simulated and measured values match well, and the maximum deviation between the simulated and measured values of the maximum velocity of the vertical slots is 4.55%, which indicates that the numerical model can simulate the hydraulic characteristics well and can be used for subsequent studies.

### 2.4. Cases

In order to discuss the influence of permeability ratio W, offset ratio R, bottom slope S and pool length *L* on flow conditions, the cases were designed, respectively; all cases are shown in Table 1. Each case keeps the side slope at 1/2 and bottom width at 4.0 m unchanged.

According to the engineering background, the design average bottom slope of the fishway is 1/250. In the study, the bottom slope is controlled to be 1/250 and the pool length is 10 m. The effects of changes in W and R on the velocity are investigated. In cases 1~6, where the vertical slots are adjacent to the bottom of the side slope on one side (i.e., a = 0 m), the permeability ratio is changed by adjusting the width of vertical slots, and the reasonable threshold of the permeability ratio is analyzed according to the results. On this basis, cases 2 and 7~11 control the permeability ratio unchanged, adjust the offset ratio by changing *a* to obtain a reasonable threshold of the offset ratio.

The bottom slope is designed according to the terrain topography, and different slopes can be used for different lots, so it is necessary to study the bottom slope. Cases 9, 12 and 13 study the effect of bottom slope change on the velocity under the control of the constant permeability ratio and offset ratio. On this basis, cases 12, 14 and 15 control the bottom slope of 1/200, to further demonstrate the reasonable threshold value of the permeability ratio. To further optimize the hydraulic characteristics, cases 12, 16 and 17 control the permeability ratio, offset ratio and bottom slope constant, and the influence of the pool length on the velocity was studied. On this basis, the pool length is further discussed.

### 2.5. Indicators and Analysis Methods

In the fishway design, the most important concern is whether fishes can successfully pass, and the most intuitive hydrodynamic information is the velocity distribution in the fishway. In this study, the maximum velocity of the control sections (i.e., the maximum velocity of the vertical slots) is mainly used as the control index. The study controls the maximum velocity of the control sections at about 1.0 m/s.

To avoid the influence of upstream and downstream on the calculation results, the maximum velocity from the 4th vertical slot to the 9th vertical slot (Figure 2 No. 1#~6#) from upstream to downstream was studied and counted. The data’s maximum, minimum and average values were analyzed as the maximum velocity of the control section. The relationship between each influencing factor and the maximum velocity of the control section was studied. At the same time, due to the uneven distribution of the velocity at the vertical slot, even if the maximum velocity exceeds the limit, there is still a certain range of channels available for fish to move up. In order to quantitatively analyze the area range of the velocity at vertical slots, the downstream edge of the vertical slot from 1# to 6# was taken as the statistical section (the measuring position is shown in Figure 1), and the distance between adjacent measuring points on each statistical section was 0.1 m. The velocity exceeding 1.0 m/s was defined as the exceeded velocity. The number of exceeded velocity points on the statistical section was counted, and the ratio of exceeded velocity points to the total number of points was the exceeded velocity ratio, which is used to approximate the area range of the exceeded velocity in the vertical slots. Considering the above indicators, the reasonable thresholds of permeability ratio and offset ratio can be concluded.

In order to explore the influence of the above influencing factor changes on the flow pattern of the pool, three analytical layers of 0.2 *h*, 0.5 *h* and 0.8 *h* (*h* is the water depth of the fishway) were divided, representing the bottom, middle and surface layers of the pool. The middle layer was used as the representative water layer to give the velocity vector distribution of the pool to analyze the flow pattern. At the same time, the study took the intermediate pool as the representative; 21 measuring sections (starting from the center section of the partition wall and ending at the center section of the next partition wall) were set up equally along the length of the pool in the bottom, middle and surface layers, respectively. A measuring point was arranged every 0.1 m on each measuring section, and the maximum velocity on each measuring section was counted as well as the lateral position of the section where it was located. The change in velocity along the way can reflect the head loss in the pool to a certain extent. It is generally believed that the better the attenuation effect of velocity along the way, the better the energy dissipation of the fishway pool. The change in the lateral position of the maximum velocity can reflect the mainstream trajectory and analyze the flow pattern in the pool. The above analysis can further demonstrate the rationality of the threshold of the permeability ratio and offset ratio.

## 3. Results and Discussion

### 3.1. Influence of Permeability Ratio on Velocity

**Figure 6:**
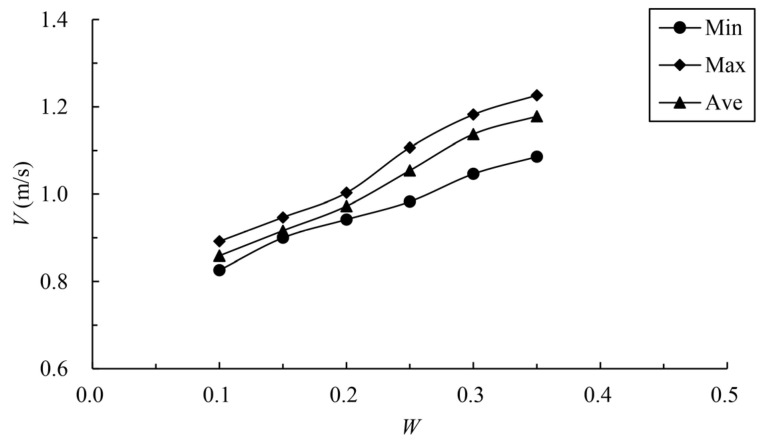
Relationship between permeability ratio and vertical-slot maximum velocity.

The calculated results of the maximum velocity of different vertical slots in the fishway with different permeability ratios are shown in Table 2 and Figure 6. It can be seen from the figure that with the increase in the permeability ratio, the minimum, maximum and average values of the maximum velocity of vertical slots show a gradually increasing trend. Based on the case of W=0.1, when the permeability ratio increases by 50%, 100%, 150%, 200% and 250%, the average maximum velocity of vertical slots increases by 6.7%, 13.25%, 22.76%, 32.44% and 37.22%, respectively. When the permeability ratio is 0.25, the maximum velocity of vertical slots reaches 1.107 m/s, the average maximum velocity reaches 1.054 m/s, and the area range of exceeded velocity is about 5%. When the permeability ratio reaches 0.30, the maximum velocity reaches 1.182 m/s and the average maximum velocity reaches 1.137 m/s; the area range of exceeded velocity is about 19%. When the permeability ratio reaches 0.35, the maximum velocity reaches 1.227 m/s and the average maximum velocity reaches 1.179 m/s; the area range of exceeded velocity is about 33%. It can be seen that the permeability ratio is the main factor affecting the maximum velocity of the control section. Even if the maximum velocity exceeds the limit, there is still a certain range of channels available for fish to move up. To meet the basic requirements, the permeability ratio of the partition wall should be no more than 0.30.

**Figure 7:**
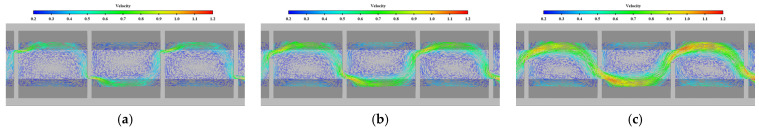
Comparison of flow patterns in the middle layer under different permeability ratios (unit: m/s). (**a**) *W* = 0.1; (**b**) *W* = 0.2; (**c**) *W* = 0.3.

Figure 7 compares the velocity vector in the middle layer under typical permeability ratio cases. As can be seen from the figure, the flow structure of each pool is relatively similar. Due to the vertical slot being arranged on the opposite side, the mainstream in the pool meanders forward. The mainstream first rushes to the side slope of the short partition wall, and then the mainstream streamline bends into the next vertical slot on the opposite side. The velocity distribution of vertical slots is uneven, and the maximum velocity is mainly located at the upper edge of the long wall and the lower edge of the short wall in contact with the mainstream. Under different cases, the mainstream of the pool is clear. There is a wide range of reflux areas on one side of the mainstream. The width of the mainstream area increases and the area of the reflux decreases with the increase in the permeability ratio. However, the velocity of the reflux area is basically less than 0.2 m/s, so the reflux is not intense. The low-velocity area can provide a good rest area for the upstream fish, and does not cause the fish to become disoriented.

**Figure 8:**
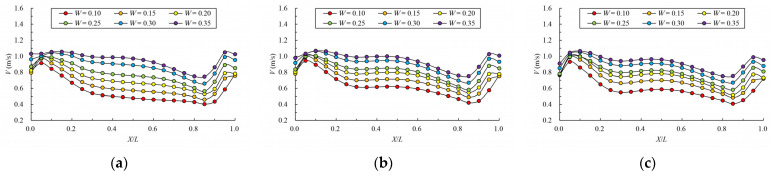
Maximum velocity attenuation under different permeability ratio. (**a**) bottom layer; (**b**) middle layer; (**c**) surface layer.

**Figure 9:**
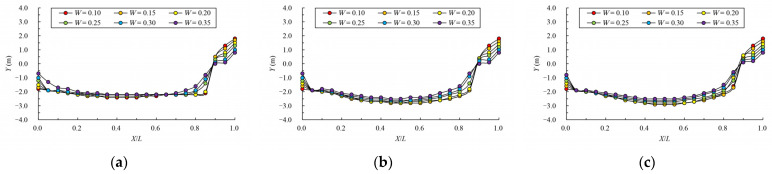
Mainstream trajectory under different permeability ratio. (**a**) bottom layer; (**b**) middle layer; (**c**) surface layer.

Figure 8 and Figure 9 give the mainstream velocity variation and mainstream trajectory variation for different analyzed water layers under different cases. X indicates the longitudinal coordinate of the section, L indicates the length of the pool, V indicates the maximum velocity of the pool, and Y represents the transverse position of the section where the maximum velocity is located (the centerline position of the pool is recorded as 0). From the variation in mainstream velocity along the flow path, the attenuation laws of velocity in different water layers are similar. The mainstream velocity decreases gradually near the control section and then increases near the next control section. The flow diffusion is sufficient, and the energy dissipation effect is good. The main flow is on one side, comparing the data from three water layers, due to the bottom layer having the smallest water area, and the main flow collides with the side slope faster; the velocity of the mainstream decays faster, so the energy dissipation is slightly better than the middle layer and surface layer. Additionally, the surface layer has the largest water area, so the water flow diffusion is more adequate, and the velocity attenuation is slightly better than the middle layer. The attenuation law of velocity under different permeability ratios is similar. When the permeability ratio is less than 0.30, the maximum velocity can be attenuated to about 0.4 m/s–0.7 m/s, but when the permeability ratio reaches 0.35, the maximum velocity can be attenuated to about 0.8 m/s. The high velocity in the pool may adversely affect the passing fish, so it is appropriate to control the permeability ratio below 0.30. From the change in mainstream trajectory, due to the vertical slot being close to the bottom of the side slope, the mainstream trajectory of each case is consistent. The mainstreams all, first, are biased to the side of the short partition wall, and then collide with the side slope and turn to the next vertical slot. The spans of mainstream trajectories flowing through are slightly different. There are different degrees of mainstream distortion, especially when the permeability ratio is small and the mainstream distortion is large, which should be avoided in the design but it can be optimized by adjusting the offset ratio.

### 3.2. Influence of Offset Ratio on Velocity

**Figure 10:**
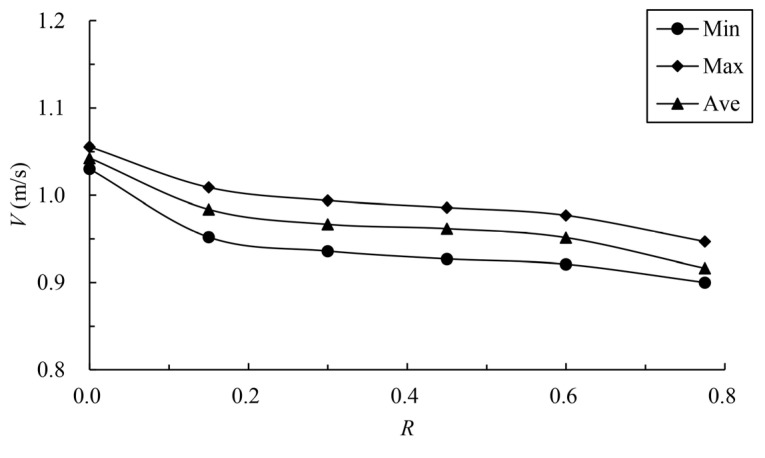
Relationship between offset ratio and vertical-slot maximum velocity.

**Figure 11:**
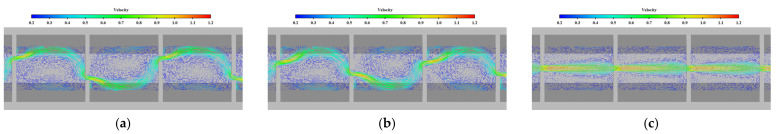
Comparison of flow patterns in middle layer under different offset ratios (unit: m/s). (**a**) *R* = 0.6; (**b**) *R* = 0.3; (**c**) *R* = 0.

**Figure 12:**
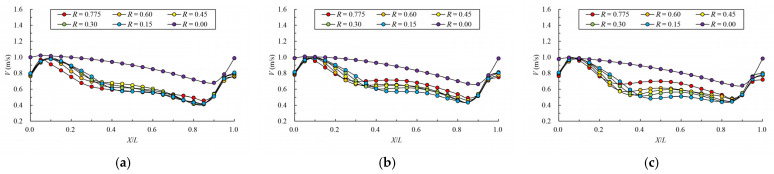
Maximum velocity attenuation under different offset ratios. (**a**) bottom layer; (**b**) middle layer; (**c**) surface layer.

**Figure 13:**
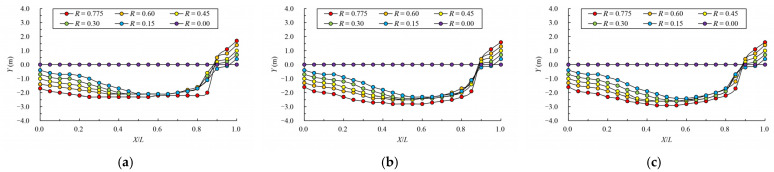
Mainstream trajectory under different offset ratios. (**a**) bottom layer; (**b**) middle layer; (**c**) surface layer.

The calculated results of the maximum velocity of different vertical slots with different offset ratios are shown in Table 3 and Figure 10. Figure 11 compares the velocity vector in the middle layer under typical offset ratio cases. The mainstream velocity variation and mainstream trajectory variation for different analyzed water layers are shown in Figure 12 and Figure 13. The results show that when the offset ratio *R* ≥ 0.15, the maximum, minimum and average values of the data for different cases are not particularly different and are roughly distributed between 0.90 m/s and 1.00 m/s. When R=0, the upstream and downstream pools form a direct jet, the maximum velocity of the vertical slot increases obviously, and the area range of exceeded velocity is about 28%. Therefore, the offset ratio should not be too small; it is recommended that R≥0.15.

When R=0, the total resistance inside the fishway is significantly reduced. The flow pattern changes, and a direct jet is formed between the vertical slots; the maximum velocity is located in the center of the jet, and the area of the reflux on both sides of the jet is basically the same. When R≥0.15, the flow pattern is similar, the mainstream is bypassing in the pool, and the velocity distribution in the pool is also similar. When R≥0.15, the variation law of the mainstream velocity is basically the same. The maximum velocity of the pool decreases from 1.0 m/s to about 0.4 m/s, and the energy dissipation is good; such a clear mainstream can attract fish for upstreaming. When *R* = 0, the maximum velocity decreases to about 0.7 m/s along the way, the energy dissipation is significantly reduced, and the direct jet flow with high velocity is maintained in the pool, which may have a negative impact on fish passing. From the mainstream trajectory changes, with the decrease in offset ratio, the mainstream trajectory tends to be smoother. Compared to the case where the vertical slot is close to the bottom of the side slope, the flow pattern has a greater improvement. The pool as a whole creates a “high velocity zone” and “low velocity zone”; the flow pattern is good, which is conducive for fish passing.

In general, in the case of similar flow patterns, the offset ratio is not the main factor affecting the maximum velocity of the control section. The offset ratio mainly controls the mainstream trajectory. With the decrease in the offset ratio, the mainstream tends towards the center of the pool, and the bending degree of the mainstream decreases. In order to avoid a large bending degree of the mainstream, it is suggested that the offset ratio is less than 0.6.

### 3.3. Influence of Bottom Slope on Velocity

**Figure 14:**
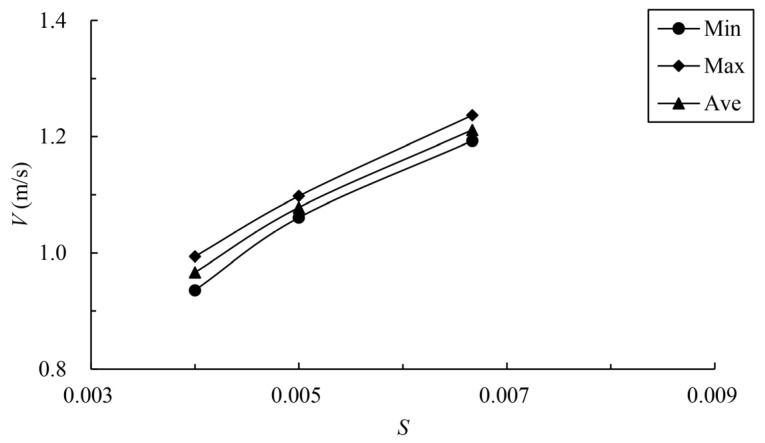
Relationship between bottom slope and vertical-slot maximum velocity.

**Figure 15:**
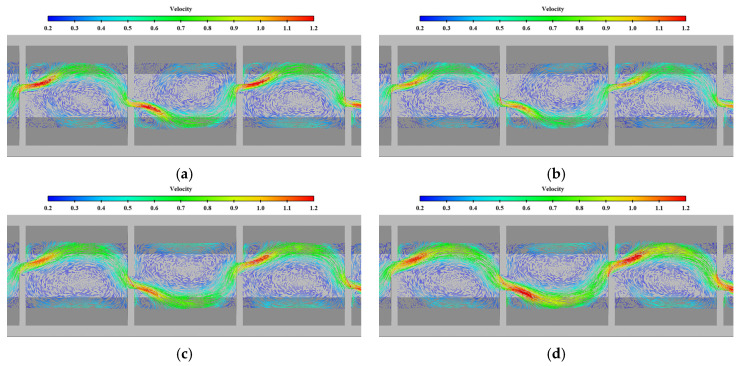
Comparison of flow patterns in middle layer under different bottom slopes (unit: m/s). (**a**) *W* = 0.15, *S* = 1/150; (**b**) *W* = 0.15, *S* = 1/200; (**c**) *W* = 0.2, *S* = 1/200; (**d**) *W* = 0.25, *S* = 1/200.

The results of maximum velocity of vertical slots with different bottom slopes under typical permeability ratio and offset ratio are shown in Table 4 and Figure 14. The velocity vectors of the pool under typical cases are shown in Figure 15. From the chart, it can be seen that under the same permeability ratio and offset ratio, with the bottom slope steepening, each index increased greatly. When W=0.15 and R=0.3, the average data at 1/250 bottom slope are 0.967 m/s, 1.079 m/s at 1/200 bottom slope (the area range of the exceeded velocity is about 22%) and 1.212 m/s at 1/150 bottom slope (the area range of exceeded velocity is about 38%). The average data of maximum velocity in vertical slots reflect the maximum velocity level of vertical slots. However, the velocity distribution at the vertical slot itself is uneven; when the bottom slope is 1/200, the area of velocity exceeding the limit is not very large, which can be considered to basically meet the requirements. When the bottom slope is 1/150, the area of velocity exceeding the limit is too large, so it does not meet the requirements. Therefore, the steep bottom slope should be avoided in the design.

**Figure 16:**
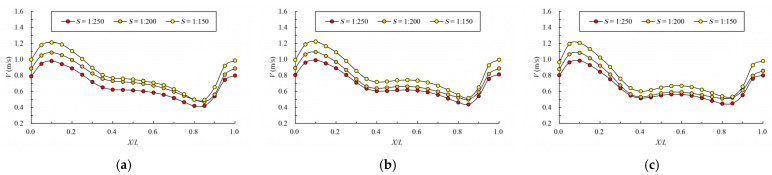
Maximum velocity attenuation under different bottom slopes. (**a**) bottom layer; (**b**) middle layer; (**c**) surface layer.

**Figure 17:**
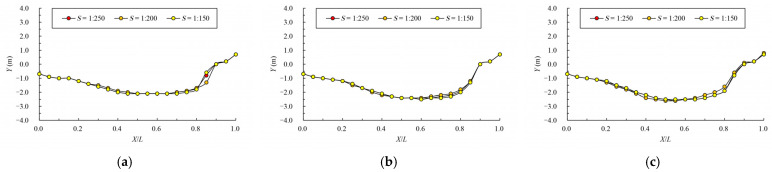
Mainstream trajectory under different bottom slopes. (**a**) bottom layer; (**b**) middle layer; (**c**) surface layer.

The variation law of mainstream velocity along the path and the variation law of mainstream trajectory are shown in Figure 16 and Figure 17. As can be seen from the chart, as the bottom slope becomes steep, the velocity of the pool increases, but the overall variation law is consistent. For mainstream trajectory, when the permeability ratio and offset ratio remain unchanged, the change in bottom slope has no effect on the mainstream trajectory.

When the bottom slope is adjusted to 1/200, the velocity in the pool increases, and the threshold of the permeability ratio needs to be further demonstrated. Keeping the offset ratio 0.3 and bottom slope 1/200 unchanged, when the permeability ratio reaches 0.2, the average datum of the maximum velocity is 1.123 m/s, and the area range of the exceeded velocity is about 23%, which can be considered to basically satisfy the requirements. When the permeability ratio rises to 0.25, the velocity at the vertical slot exceeds the standard in a large range, so this case does not satisfy the requirements. Comprehensively, the bottom slope is the key design parameter affecting the velocity of the control section of the fishway. When the bottom slope is adjusted to 1/200, the permeability ratio and offset ratio must be reconsidered. It is recommended to control the permeability ratio below 0.20 and the offset ratio between 0.30 and 0.60.

### 3.4. Influence of Pool Length on Velocity

**Figure 18:**
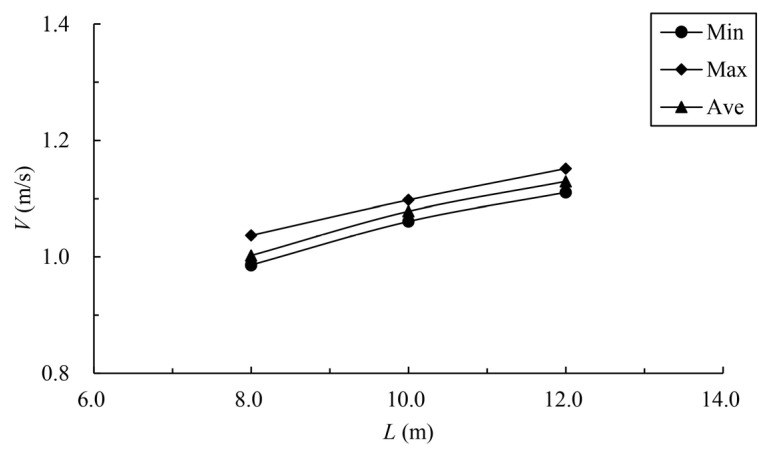
Relationship between pool length and vertica- slot maximum velocity.

**Figure 19:**
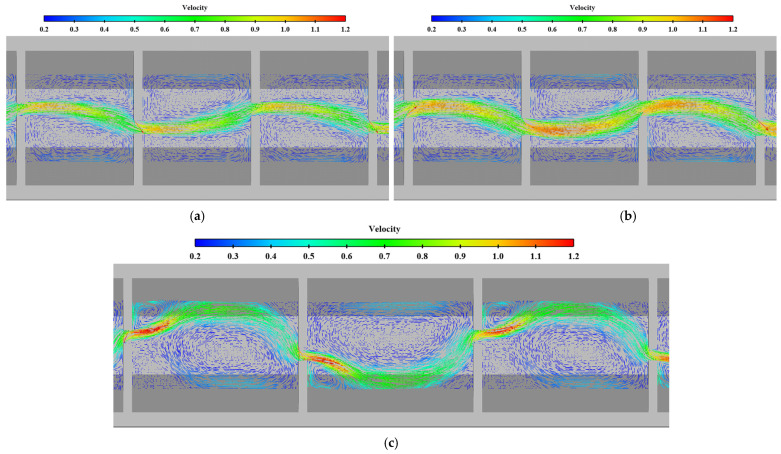
Comparison of flow patterns in middle layer under different pool lengths (unit: m/s). (**a**) *W* = 0.15, *L* = 8 m; (**b**) *W* = 0.2, *L* = 8 m; (**c**) *W* = 0.15, *L* = 12 m.

When the bottom slope is 1/200, the results of maximum velocity of vertical slots with different pool length under the typical permeability ratio and offset ratio are shown in Table 5 and Figure 18. The velocity vectors under typical cases are shown in Figure 19. It can be seen from the chart that the maximum, minimum and average data increase with the increase in the pool length under the same permeability ratio and offset ratio. The reason is that when the bottom slope and the water depth of the pool are fixed, the longer the length of the pool, and the greater the head consumed by the unit pool. When W=0.2, R=0.3 and L=10 m, the average datum is 1.123 m/s. When the length is adjusted to 8 m, the average datum decreases to 1.088 m/s; the area range of the exceeded velocity is about 21%, so the exceeding range is not very large, which can be considered to basically satisfy the requirements. When the permeability ratio decreases to 0.15, the average datum is reduced to 1.002 m/s, which can better meet the requirements.

**Figure 20:**
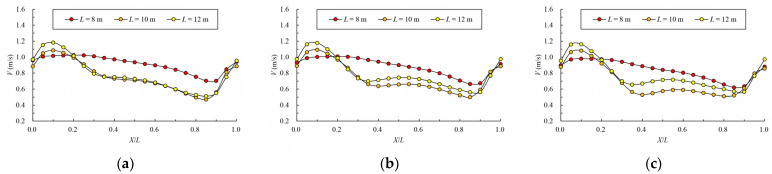
Maximum velocity attenuation under different pool lengths. (**a**) bottom layer; (**b**) middle layer; (**c**) surface layer.

**Figure 21:**
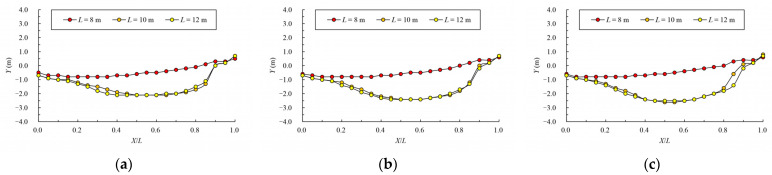
Mainstream trajectory under different pool lengths. (**a**) bottom layer; (**b**) middle layer; (**c**) surface layer.

The variation law of mainstream velocity along the path and the variation law of the mainstream trajectory are shown in Figure 20 and Figure 21. It can be seen from the chart that when the length of the pool is 10 m and 12 m, the mainstream is biased towards one side, and the attenuation law of the velocity and the mainstream trajectory is roughly the same. The velocity in the fishway pool can quickly decay to about 0.4 m/s, so the energy dissipation efficiency is high. When the length of the pool is adjusted to 8 m, the flow pattern in the pool changes, which is similar to the “direct jet”. In this case, the mainstream tends towards the center of the pool and bypasses within the pool. The mainstream is less affected by the side slope, so the attenuation of velocity is reduced, with the maximum velocity decaying to about 0.6 m/s in the surface layer and the maximum velocity being slightly higher in the middle and bottom layers. Although the energy dissipation efficiency in the pool is reduced, the mainstream trajectory is smoother, which is conducive to the fish to find the mainstream to upstream more quickly. Therefore, the study considers that the shortening of the pool length to 8 m slightly improves the flow pattern, and suggests optimizing the pool length to 8 m.

### 3.5. The Field Applications

The design parameters such as the permeability ratio, offset ratio, bottom slope and pool length can be used to optimize the flow conditions of the nature-like fishway and provide guidance for fishway design. Taking into account the results from 3.1~3.4, the recommended design parameters’ thresholds for the Mopiling nature-like fishway are: when the bottom slope of the fishway is 1/250 and the pool length is 10 m, the permeability ratio should not be greater than 0.30 and the offset ratio should be located between 0.15 and 0.60. When the bottom slope is adjusted to 1/200, it is recommended that the permeability ratio is below 0.20, the offset ratio between 0.30 and 0.60, and the pool length can be adjusted to 8 m.

For the field applications, the fishway structural arrangement should be adjusted within the above-given design parameter thresholds. The bottom slope is designed according to the terrain topography, and different slopes can be used for different lots. The cross-section can be regular or irregular, and natural materials such as stones, gabions and vegetation can be used to make it fit with the natural river as much as possible. Due to the complexity of the problem, the tested model in the study is a simplified and generalized model of a nature-like fishway; specific details of natural optimization and the other structures in the nature-like fishway such as bends, shoals and deep pools need to be further studied. To date, since there is a lack of sufficient data about the post-evaluation of the fish-passing effect in China, it is valuable to carry out prototype observations to evaluate the efficiency of fish passing.

## 4. Conclusions

(1) The study simplifies and generalizes the irregular nature-like fishway based on two important causes of flow energy consumption in a nature-like fishway: water blockage by the partition wall and mainstream bypass deflection of the pool. The water-permeable area of the partition wall is expressed by the permeability ratio of the control section, and the mainstream deflection length of the pool is expressed by the offset ratio of the control section of the adjacent pool. Combining the bottom slope and pool length, the effect of the above parameters on the flow structure is investigated through a 3D turbulent numerical simulation, and the design parameter thresholds satisfying the requirements are summarized to provide guidance for fishway design. The relevant design parameters and optimization strategies can provide a reference for similar projects.
(2) The study showed that there are two basic flow patterns in the fishway pool: the mainstream is biased to one side and the mainstream tends towards the center. Under the same conditions, when the offset ratio is smaller and the pool length is shorter, the mainstream tends towards the center of the pool, and vice versa, the mainstream is biased to one side. Different flow patterns have a great influence on the velocity of the control section, the attenuation law of the main flow velocity and the main flow trajectory. The attenuation law of the main flow velocity and the main flow trajectory are basically the same under the same flow pattern.
(3) Permeability ratio, bottom slope and pool length are the main factors affecting the maximum velocity of the control section. Under the same conditions, the maximum velocity of the control section increases with the increase in permeability ratio, bottom slope and pool length. The offset ratio has limited effect on the maximum velocity of the control section. Under the same conditions, the offset ratio decreases, the velocity of the control section only slightly increases, and the mainstream becomes smoother.
(4) Within the recommended design parameter thresholds for the Mopiling nature-like fishway, the maximum velocity at the vertical slot can be basically controlled at about 1.0 m/s. The velocity distribution at the vertical slot is uneven, so that even if the maximum velocity exceeds 1.0 m/s, there is still a sufficient area available for upstream. The mainstream in the pool is clear and the flow pattern with the “high velocity zone” and “low velocity zone” is good, which can basically satisfy the requirements of fish passing.

## Figures and Tables

**Table 1:** Numerical model calculation cases.

Case	*b* (m)	*W*	*a* (m)	*R*	*S*	*L* (m)
1	0.6	0.1	0	0.85	1/250	10
2	0.9	0.15	0	0.775	1/250	10
3	1.2	0.2	0	0.7	1/250	10
4	1.5	0.25	0	0.625	1/250	10
5	1.8	0.3	0	0.55	1/250	10
6	2.1	0.35	0	0.475	1/250	10
7	0.9	0.15	0.35	0.6	1/250	10
8	0.9	0.15	0.65	0.45	1/250	10
9	0.9	0.15	0.95	0.3	1/250	10
10	0.9	0.15	1.25	0.15	1/250	10
11	0.9	0.15	1.55	0	1/250	10
12	0.9	0.15	0.95	0.3	1/200	10
13	0.9	0.15	0.95	0.3	1/150	10
14	1.2	0.2	0.8	0.3	1/200	10
15	1.5	0.25	0.65	0.3	1/200	10
16	0.9	0.15	0.95	0.3	1/200	8
17	0.9	0.15	0.95	0.3	1/200	12
18	1.2	0.2	0.8	0.3	1/200	8

**Table 2:** Maximum velocity of vertical slots under different permeability ratios.

W	b (m)	a (m)	Maximum Velocity of Vertical Slots V (m/s)								
			1#	2#	3#	4#	5#	6#	Min	Max	Ave
0.10	0.60	0	0.868	0.858	0.892	0.842	0.867	0.826	0.826	0.892	0.859
0.15	0.90	0	0.901	0.947	0.909	0.937	0.904	0.900	0.900	0.947	0.916
0.20	1.20	0	0.942	0.986	0.959	1.003	0.984	0.962	0.942	1.003	0.973
0.25	1.50	0	0.983	1.022	1.036	1.081	1.107	1.097	0.983	1.107	1.054
0.30	1.80	0	1.046	1.125	1.182	1.145	1.158	1.168	1.046	1.182	1.137
0.35	2.10	0	1.086	1.170	1.185	1.215	1.188	1.227	1.086	1.227	1.179

**Table 3:** Maximum velocity of vertical slots under different offset ratios.

W	a (m)	R	Maximum Velocity of Vertical Slots V (m/s)								
			1#	2#	3#	4#	5#	6#	Min	Max	Ave
0.15	0.00	0.775	0.901	0.947	0.909	0.937	0.904	0.900	0.900	0.947	0.916
0.15	0.35	0.600	0.954	0.977	0.958	0.957	0.921	0.942	0.921	0.977	0.952
0.15	0.65	0.450	0.934	0.927	0.983	0.986	0.959	0.981	0.927	0.986	0.962
0.15	0.95	0.300	0.936	0.967	0.968	0.994	0.966	0.969	0.936	0.994	0.967
0.15	1.25	0.150	0.952	1.002	0.983	0.994	1.009	0.963	0.952	1.009	0.984
0.15	1.55	0.000	1.056	1.053	1.043	1.030	1.036	1.038	1.030	1.056	1.043

**Table 4:** Maximum velocity of vertical slot under different bottom slopes.

W	R	S	Maximum Velocity of Vertical Slots V (m/s)								
			1#	2#	3#	4#	5#	6#	Min	Max	Ave
0.15	0.30	1:250	0.936	0.967	0.968	0.994	0.966	0.969	0.936	0.994	0.967
0.15	0.30	1:200	1.061	1.072	1.079	1.073	1.098	1.088	1.061	1.098	1.079
0.15	0.30	1:150	1.227	1.218	1.197	1.237	1.193	1.198	1.193	1.237	1.212
0.20	0.30	1:200	1.074	1.102	1.141	1.131	1.160	1.132	1.074	1.160	1.123
0.25	0.30	1:200	1.128	1.203	1.249	1.238	1.274	1.257	1.128	1.274	1.225

**Table 5:** Maximum velocity of vertical slot under different pool lengths.

W	R	S	L (m)	Maximum Velocity of Vertical Slots V (m/s)								
				1#	2#	3#	4#	5#	6#	Min	Max	Ave
0.15	0.30	1:200	8.0	0.986	1.012	1.037	0.987	1.002	0.990	0.986	1.037	1.002
0.15	0.30	1:200	10.0	1.061	1.072	1.079	1.073	1.098	1.088	1.061	1.098	1.079
0.15	0.30	1:200	12.0	1.139	1.152	1.126	1.137	1.115	1.111	1.111	1.152	1.130
0.20	0.30	1:200	8.0	1.106	1.140	1.071	1.077	1.049	1.086	1.049	1.140	1.088
0.20	0.30	1:200	10.0	1.074	1.102	1.141	1.131	1.160	1.132	1.074	1.160	1.123

## References

[1] Chen K., Chang Z., Cao X., Ge H. (2012). Status and prospection of fish pass construction in China. J. Hydraul. Eng..

[2] Wu X., Shi J. (2014). Construction and management of fish passage on Shaliu River adjacent to Qinghai Lake based on ecological restoration. Trans. Chin. Soc. Agric. Eng..

[3] Peng W., Liu X., Wang Y., Zou X. (2018). Review and prospect of progress in water environment and water ecology research. J. Hydraul. Eng..

[4] Zhang Y., He Z., He Y., Zhang J., Zhang K. (2017). Analysis on the efficiency of fishway for the low-head gate dam. J. Hydraul. Eng..

[5] Daneshvar F., Nejadhashemi A.P., Woznicki S.A., Herman M.R. (2017). Applications of computational fluid dynamics in fish and habitat studies. Ecohydrol. Hydrobiol..

[6] Mao X. (2018). Review of fishway research in China. Ecol. Eng..

[7] Marriner B.A., Baki A.B.M., Zhu D.Z., Cooke S.J., Katopodis C. (2016). The hydraulics of a vertical slot fishway: A case study on the multi-species Vianney-Legendre fishway in Quebec, Canada. Ecol. Eng..

[8] Marriner B.A., Baki A.B.M., Zhu D.Z., Thiem J.D., Cooke S.J., Katopodis C. (2014). Field and numerical assessment of turning pool hydraulics in a vertical slot fishway. Ecol. Eng..

[9] Córdoba F., Fuentes J.F., Valbuena-Castro J., Azagra A., Sanz-Ronda F.J. (2021). Turning Pools in Stepped Fishways: Biological Assessment via Fish Response and CFD Models. Water.

[10] Xu T., Sun S. (2009). Numerical simulation of the flow structure in vertical slot fishway. J. Hydraul. Eng..

[11] An R., Li J., Liang R., Tuo Y. (2016). Three-dimensional simulation and experimental study for optimising a vertical slot fishway. J. Hydro-Environ. Res..

[12] Ballu A., Pineau G., Calluaud D., David L. Influence of Macro-Roughnesses on Vertical Slot Fishways. Proceedings of the 7th International Symposium on Hydraulic Structures.

[13] Bombač M., Četina M., Novak G. (2017). Study on flow characteristics in vertical slot fishways regarding slot layout optimization. Ecol. Eng..

[14] Li Y., Wang X., Xuan G., Liang D. (2019). Effect of parameters of pool geometry on flow characteristics in low slope vertical slot fishways. J. Hydraul. Res..

[15] Xia W., Gao Z., Shi X., Tan J., Luo K., Chen X. (2017). Three-demensional numerical simulation of hydraulic characteristics of vertical slot fishway. Water Resour. Power.

[16] Fuentes-Perez J.F., Silva A.T., Tuhtan J.A., Garcia-Vega A., Carbonell-Baeza R. (2018). 3D modelling of non-uniform and turbulent flow in vertical slot fishways. Environ. Model. Softw..

[17] Wei Y., Luo K., Tan J., Tang L., Wang J. (2020). Study on hydraulic characteristics of vertical slot fishway based on three turbulence models. Water Resour. Power.

[18] Roth M.S., Jhnel C., Stamm J., Schneider L.K. (2021). Turbulent eddy identification of a meander and vertical-slot fishways in numerical models applying the IPOS-framework. J. Ecohydraulics.

[19] Kirk M., Caudill C., Syms J., Tonina D. (2017). Context-dependent responses to turbulence for an anguilliform swimming fish, Pacific lamprey, during passage of an experimental vertical-slot weir. Ecol. Eng..

[20] Tan J., Gao Z., Dai H., Shi X. (2017). The correlation analysis between hydraulic characteristics of vertical slot fishway and fish movement characteristics. J. Hydraul. Eng..

[21] Yang P., Tan J., Gao Z., Dai H., Shi X., Huang T. (2018). The analysis of fish movement behavior in vertical slot fishway based on video tracking. Acta Hydrobiol. Sin..

[22] Zhang H., Li G., Han Y., Sun S., Liu H., Zheng T., Zhao H. (2020). Study on migration behaviors of juvenile *Schizothorax prenanti* in a vertical slot fish-way model. Water Resour. Hydropower Eng..

[23] Syms J., Kirk M., Caudill C., Tonina D. (2021). A biologically based measure of turbulence intensity for predicting fish passage behaviours. J. Ecohydraulics.

[24] Gao Z., Andersson H.I., Dai H., Jiang F., Zhao L. (2016). A new Eulerian–Lagrangian agent method to model fish paths in a vertical slot fishway. Ecol. Eng..

[25] Jiang Y., Yang Z., Shi X., Wu L., Nie L., Wei Y. (2018). The simulation of fish migratory trajectory in a vertical slot fishway based on multi-hydraulic indices. Chin. J. Ecol..

[26] Jin Z., Ma W., Zhang Y., Chen M., Tan J., Shi X. (2018). Assessing the swimming ability and performance of Schizothorax oconnori to cross velocity barriers in fishway. J. Hydraul. Eng..

[27] Cai L., Hou Y., Jin Y., Yang Z., Hu W., Chen X., Chen J., Huang Y., Han D. (2021). Response of fish swimming ability to body length and its application in fishway design. Trans. Chin. Soc. Agric. Eng..

[28] Shi X., Chen Q., Huang Y., Liu D., Zhuang P. (2011). Review on the methods to quantify fish’s ability to cross velocity barriers in fish passage. Acta Ecol. Sin..

[29] Gustafsson S., Österling M., Skurdal J., Schneider L.D., Calles O. (2013). Macroinvertebrate colonization of a nature-like fishway: The effects of adding habitat heterogeneity. Ecol. Eng..

[30] Dodd J.R., Cowx I.G., Bolland J.D. (2017). Efficiency of a nature-like bypass channel for restoring longitudinal connectivity for a river-resident population of brown trout. J. Environ. Manag..

[31] Song W., Xu Q., Fu X., Wang C., Pang Y., Song D. (2019). EFDC simulation of fishway in the Diversion Dahaerteng River to Danghe Reservoir, China. Ecol. Indic..

[32] FAO, DVWK (2002). Fish passes—Design, dimensions and monitoring.

[33] Li S., Ding X., Liu D. (2014). Overview of nature-simulating fishway. Yangtze River.

[34] Acharya M., Kells J., Katopodis C. (2000). Some Hydraulic Design Aspects of Nature-Like Fishways. Proceedings of the Joint Conference on Water Resource Engineering and Water Resources Planning and Management.

[35] He Y., An R., Li J., Yi W., Zeng Z. (2016). Hydraulic characteristics of nature-like fishways of rock-ramp type. J. Hydroelectr. Eng..

[36] Baki A.B.M. Numerical Modelling of Rock-Weir Type Nature-Like Fishpasses; In Proceedings of the Leadership in Sustainable Infrastructure, Vancouver, Canada, 31 May–3 June 2017.

[37] Zhang Y., Hu X., Yang F., Lv W., Zhang J., Mo W. (2021). Experimental study on structure improvement and hydraulic characteristics of artificial natural-like fishway. Yangtze River.

[38] Hu Q., Zhu L. (2020). Numerical research on the flows in nature-like fishway combined with slot and orifice. China Rural. Water Hydropower.

[39] Ma W., Liang Y., Wang M., Feng M. (2020). Study on hydraulic characteristics of vertical slot-surface outlet combined nature-imitated fishway. Water Resour. Hydropower Eng..

[40] Li G., Sun S., Liu H., Zhang C., Zhao G., Zheng T. (2017). Improving effect of hydraulic characteristics of nature-like fishway with pools and cobblestone weirs. Trans. Chin. Soc. Agric. Eng..

[41] Xu J., Wang X., Xuan G., Zheng F., Huang Y. (2017). Physical model test study on nature-like fishways. Adv. Water Sci..

[42] Zhu L., Hu Q., Wang C., Wang X. (2022). Numerical Simulation and Optimization of Flow Conditions in Zongyang Nature-like Fishway. China Rural. Water Hydropower.

